# Individualized Evaluation on Suspicion of Fibrotic Metabolic-Dysfunction-Associated Steatohepatitis: Real-World Experience from a Referral Center in Denmark

**DOI:** 10.3390/jpm16020095

**Published:** 2026-02-06

**Authors:** Eva Efsen Dahl, Gro Linno Willemoe, Mark Berner-Hansen, Frank Vinholt Schiødt

**Affiliations:** 1 Digestive Disease Center K, Bispebjerg University Hospital of Copenhagen, 2400 Copenhagen, Denmark; eva.efsen.dahl.01@regionh.dk (E.E.D.); mark.berner-hansen@regionh.dk (M.B.-H.); 2 Department of Pathology, Rigshospitalet, 2100 Copenhagen, Denmark; gro.linno.willemoe.02@regionh.dk

**Keywords:** metabolic-dysfunction-associated steatohepatitis, MASH, real-world setting, cohort study, liver biopsy, personalized medicine

## Abstract

**Background/Objectives:** New guidelines for management of metabolic-dysfunction-associated steatotic liver disease (MASLD) patients recommend an individualized medicine approach mainly targeting patients with fibrotic metabolic-dysfunction-associated steatohepatitis (MASH) and metabolic risk factors for progression of disease. This cohort study reports real-world experience for the individual evaluation and final diagnosis of patients on suspicion of fibrotic MASH according to standardized international criteria. We aimed to identify patients with significant fibrosis (F2–F4). **Methods:** Adult patients with metabolic syndrome and/or elevated alanine aminotransferases (ALT > 50) referred in a 5-year period (2018–2022) on suspicion of fibrotic MASH were included. Medical history, anthropometric measurements, and routine (blood tests, ultrasound) and specific examinations were applied. Liver biopsy was offered for definite diagnosis and to evaluate MASLD characteristics. Patient demographics and characteristics as well as the absolute number and proportion of patients with definite MASLD and fibrotic MASH are reported. **Results:** A total of 137 adult patients were included. Ten percent of patients were evaluated without liver biopsy and diagnosed with chronic liver diseases other than MASLD. Liver-biopsied patients (n = 123) had a mean age (SD) of 49 (14) years, and 50% were males. Overweight or obesity was present in 94%, dyslipidemia in 74%, hypertension in 40%, and type 2 diabetes mellitus in 34%. Of all 137 patients, 104 (76%) were diagnosed with definite MASLD and 80 (58%) with definite MASH. A total of 74 (54%) patients had definite fibrotic MASH, while 41 (30%) had significant (F2–4) fibrotic MASH. Eight patients (6%) had cirrhotic (F4) MASH. A multivariate logistic regression analysis indicated that patients with type 2 diabetes, older age, and higher BMI were associated with an apparent increased risk of F2–F4 fibrosis. **Conclusions:** The majority of referred patients had cardiometabolic–hepatic metabolic risk factors and were diagnosed with definite MASLD. More than half of these were diagnosed with fibrotic MASH. Older age, type 2 diabetes, and higher BMI were apparent risk factors for MASH F2–F4 fibrosis. We conclude that the individual cardiovascular–hepatic risk profile applied supports the new guidelines and may be useful for referral and further evaluation at expert care centers in a real-world setting.

## 1. Introduction

Metabolic-dysfunction-associated steatotic liver disease (MASLD) and metabolic-dysfunction-associated steatohepatitis (MASH) have replaced the terms non-alcoholic fatty liver disease (NAFLD) and non-alcoholic steatohepatitis (NASH) as nomenclatures for diseases associated with steatotic liver disease (SLD) and cardiometabolic risk factors [1]. MASLD covers the whole spectrum of steatotic liver disease. In some patients, hepatocyte injury can cause the development of MASH—which again can progress to fibrotic MASH. Fibrosis is the strongest predictor of long-term liver complications such as cirrhosis and hepatocellular carcinoma.

MASLD is a multifaceted disorder resulting from complex interactions with various genetic, cardiometabolic, and environmental risk factors [2]. It is now the most prevalent chronic liver disease (CLD), with a prevalence of more than 30%, still rising in parallel with the increase in the prevalence of obesity, as well as cardiovascular and metabolic comorbidity (type 2 diabetes mellitus, dyslipidemia, hypertension), which are risk factors for MASLD and progression of disease [3,4].

New guidelines for management of MASLD recommend an individualized medicine approach targeting mainly patients with fibrotic MASH and risk factors for progression of disease to liver cirrhosis [3,5]. As such, identifying patients with fibrotic MASH is eminent for prevention and management of fibrotic MASH-related complications. However, identification of fibrotic MASH at-risk patients is challenging in the real-world routine-practice setting.

In this paper, we report real-world evidence from evaluating a cohort of patients, with cardiometabolic risk factor profile, referred on suspicion of fibrotic MASH. We identified admission criteria that could help identifying fibrotic MASH. The primary outcome of the study was the proportion of patients diagnosed with definite MASLD and definite fibrotic MASH. We summarize the main findings and challenges of this approach and discuss its potential clinical implications with the new management guidelines for applying individualized medicine. Our aim was to provide real-world data for the diagnosis of and to identify possible risk factors for significant fibrotic MASH.

## 2. Materials and Methods

Adult patients were referred over a 5-year period (January 2018 through December 2022) from the primary sector and hospital departments on suspicion of MASLD or other CLD. Patients had elements of the metabolic syndrome, with or without persistently elevated alanine transaminase (ALT) liver function test levels. Patients were admitted from primary care or from specialist clinics with 2 or 3 of the following criteria: persistent ALT > 50 U/L, presence of liver steatosis on ultrasound (US) or CT scan, and type 2 diabetes.

**Figure 1:**
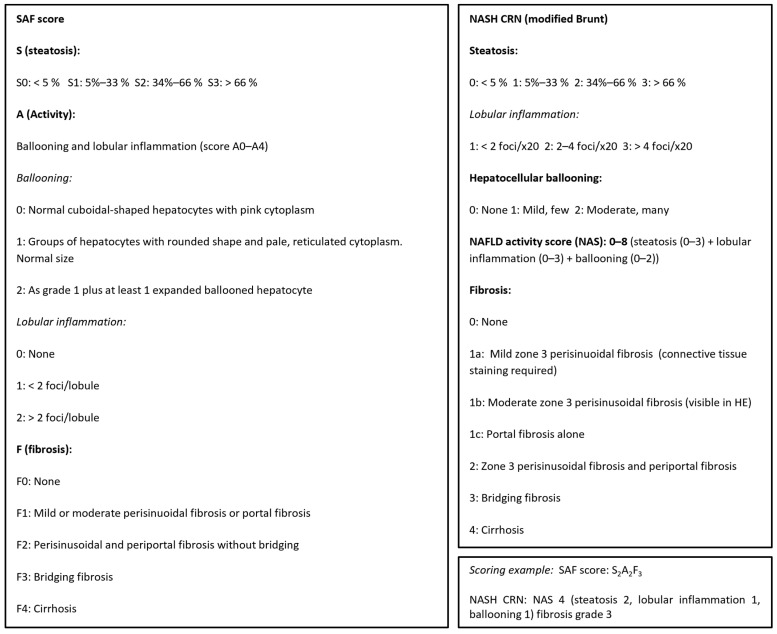
Liver biopsies were histologically scored according to the NASH (non-alcoholic steatohepatitis), CRN, and SAF (steatosis–activity–fibrosis) scoring systems, adapted from [6,7,8].

**Figure 2:**
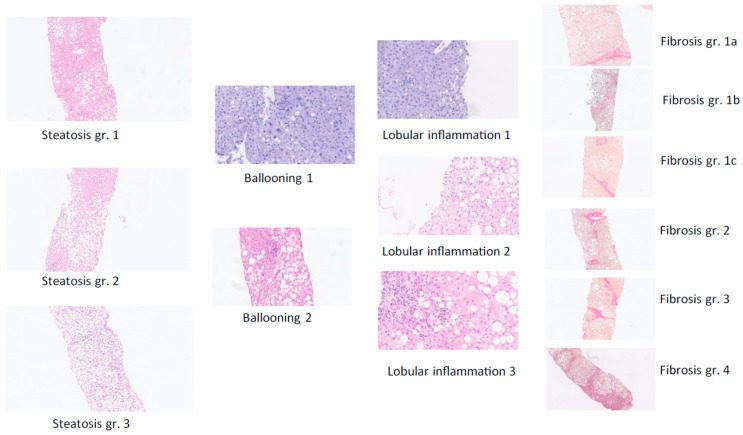
The diagnosis of definite MASLD and definite fibrotic MASH was based on liver-biopsy histopathology based on the sum of scores for steatosis, hepatocellular ballooning, and lobular inflammation (haemotoxylin and eosin staining) as well as the degree of fibrosis from F0 (no fibrosis) to F4 (cirrhosis) per regulatory CRN criteria [6]—see figure C—complemented by the SAF score, which is useful to assess the presence of active steatohepatitis (MASH) versus simple steatotic liver disease [8]. Examples from the present study of histological scoring of steatosis grade, hepatocyte damage with ballooning, lobular inflammation, and fibrosis stage per NASH CRN classification are shown [6].

All patients were evaluated using standardized general work-up and an individualized (case-by-case) approach. Initial blood tests included liver function tests and metabolic markers. Differential diagnoses to MASLD were carefully evaluated. Alcohol and drug use was recorded, and chronic viral hepatitis, autoimmune liver disease, hemochromatosis, drug-induced liver disease, and lesser common causes of liver disease were evaluated. The diagnostic imaging tool consisted of abdominal ultrasound (US) or sometimes CT scan. Transabdominal US-guided liver biopsy was offered, and after informed consent, liver biopsy was performed for the diagnosis of definite fibrotic MASH. We required platelets > 40 × 10^9^ cells/L, INR < 1.5, hemoglobin > 5.0 mmol/L, and a normal APTT before biopsy was performed. Biopsies were assessed by the same experienced histopathology expert (co-author GLW) for definite diagnosis according to international regulatory recommendations in a standardized manner [6] (for further details, see Figure 1 and Figure 2). Diagnosis of MASLD, MASH, or fibrotic MASH was made according to standard criteria [6,7,8]. We reviewed the gathered patient data for the final diagnosis. Data are reported as mean (+/− SD). SPSS 29.0.1.0 (IBM) software was used for statistical analyses.

The study was conducted according to the guidelines of the Declaration of Helsinki and approved by the Institutional Review Board of Region Hovedstaden, Center for Sundhed, Copenhagen, Denmark (jr-nr R-24067899, approval date 13 January 2025). Informed consent was obtained from all subjects involved in the study. The dataset is available on request from the authors. Data were collected retrospectively.

## 3. Results

### 3.1. Referred Patients

A total of 137 patients (all-comers) were referred for evaluation. Mean age (SD) was 48 (15) years, and 68 (50%) were men. Mean body weight was 93 (20) kg, and BMI was 31 (9) kg/m^2^.

**Figure 3:**
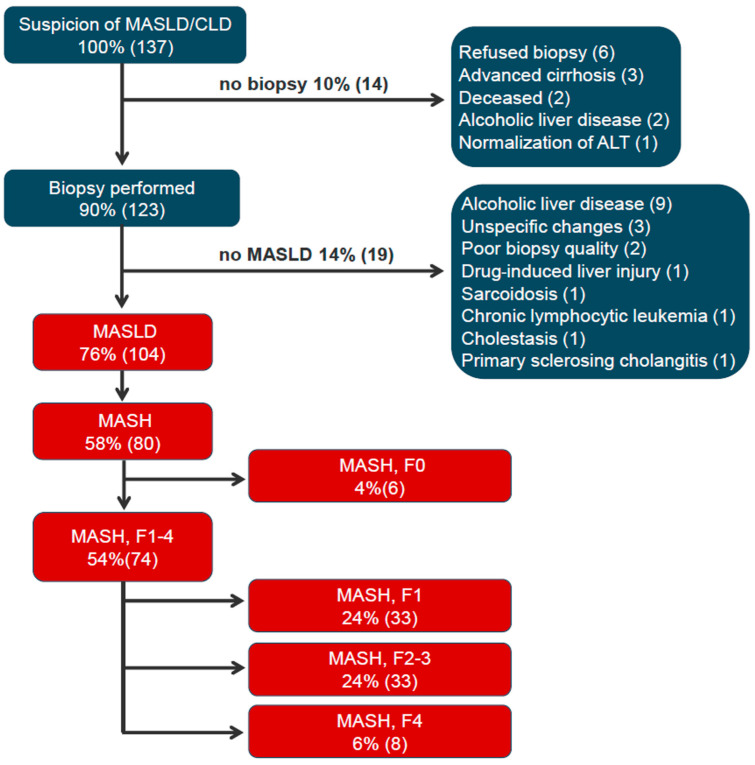
Diagnostic flow diagram of patients referred on suspicion of MASLD. Flow chart depicts the patient work-up and diagnosis of the 137 patients referred on the suspicion of MASLD. Patients are represented in % of the total 137 patients and numbers (n). Abbreviations: CLD: chronic liver disease; MASLD, metabolic-dysfunction-associated steatotic liver disease; ALT, alanine transaminase; MASH, metabolic-dysfunction-associated steatohepatitis; F0 (no fibrosis); F1, F2, F3, and F4 (cirrhosis) [6].

Figure 3 depicts the diagnostic work-up flow diagram. All non-MASLD patients had ALT > 50 U/L. All 123 liver-biopsied (90% of all-comers, 123/137) met cardiometabolic comorbidity and risk factor profile as per MASLD definition [1]. A total of 104 patients (76%) had definite MASLD by liver histopathology, whereas 80 patients (58%) had definite MASH.

Only 1 of the 123 liver-biopsied patients experienced an adverse event related to liver biopsy, which was a minor intraabdominal bleeding, which resolved spontaneously.

### 3.2. MASLD Patients

For the 104 patients diagnosed with definite MASLD, mean age was 49 (14) years, 53 (51%) were men, and 78 (75%) were Caucasians. Mean body weight was 95 (19) kg, and mean BMI was 32 (5) kg/m^2^; normal BMI was present in 6 (6%), overweight (≥25 kg/m^2^) in 98 (94%), and obesity (≥30 kg/m^2^) in 62 (60%) of patients. ALT was >50 U/L in 85 of 104 patients (82%).

Comorbidities included dyslipidemia in 78 (75%), hypertension in 42 (40%), type 2 diabetes mellitus in 36 (35%), hypothyroidism in 11 (11%), and elevated ALT > 50 U/L in 85 (82%) of patients. A total of 80 (77%) of the MASLD patients had definite MASH, 74 (71%) had definite fibrotic MASH, and 41 (39%) had definite clinically significant fibrotic (F2–F4) MASH. A total of 29 patients had F2 fibrosis, 4 patients had F3, and 8 patients were cirrhotic (F4, see Figure 3).

**Figure 4:**
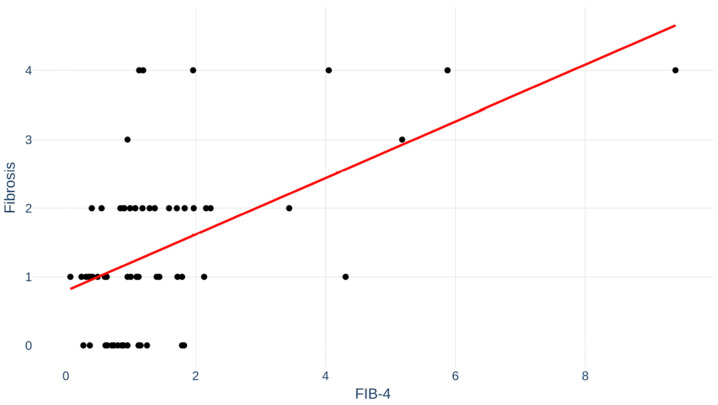
Fibrosis index for liver fibrosis (FIB-4) for patients correlated to fibrosis stage. Pearson’s r = 0.53; *p* < 0.001.

Fibrosis index for liver fibrosis (FIB-4) score, a non-invasive test for fibrosis, was available in 64 (62%) of the MASLD patients. Correlation between FIB-4 and degree of fibrosis is depicted in Figure 4. Pearson’s r was 0.53; *p* < 0.001.

A multivariate logistic regression analysis for the prediction of significant fibrosis (F2–F4) is presented in Table 1. Older age, the presence of diabetes type 2, (and higher BMI) were apparent risk factors for F2–F4 fibrosis.

## 4. Discussion

The present study provides real-world evidence supporting that a majority of patients with guideline-recognized individual risk profile (metabolic syndrome and abnormal liver function tests) for advance liver disease, admitted to our outpatient clinic, were diagnosed with definite fibrotic MASH, which requires further multi-disciplinary management.

The prevalence of fibrotic MASH and related incidence of hepatic decompensation, hepatic and non-hepatic cancers, and overall death (mainly due to major cardiovascular events) is expected to continue increasing over the next decades. Accordingly, identifying patients with MASH, who are at high risk of developing significant hepatic fibrosis, is crucial for the prevention and management of liver-related complications [1].

Individualized medicine is integrated into the new guidelines for management of MASLD. Accordingly, a tailored individual approach is recommended based on the subject’s demographics and characteristics, including comorbidities and various biomarkers (e.g., hepatic function tests, fibrosis, histopathology, and imaging) associated with disease pathogenesis [2,3,4,5]. The diagnosis of definite MASH and staging fibrosis in the real-world setting still poses considerable challenges. Although non-invasive diagnostics (blood indices alone or in combination with imaging modalities) can provide information on the entire liver, no histological characteristics can be assessed adequately. Only liver biopsy allows for an assessment of microscopic features (ballooning, lobular inflammation, Mallory bodies, micro-vesicular vs. macro-vesicular steatosis, non-alcoholic fatty liver disease activity score (NAS), accurate staging of fibrosis) [9,10] and to rule out or affirm coexistence of other forms of CLD [3]. As such, histopathology by liver biopsy is still the gold standard for *definite* diagnosis, while non-invasive diagnostics are useful for *possible* diagnosis. Most patients in the present study accepted liver biopsy, allowing the best possible evaluation and definite diagnosis of their CLD.

The vast majority (76%) of the patients evaluated with liver biopsy in the present real-world study were diagnosed with definite MASLD, and the majority of these (71%) with fibrotic MASH. Evaluated patients had expected features of the metabolic syndrome with cardiometabolic comorbidity risk profile for progression of MASLD. Data from the present study are in line with another real-world study [11], which demonstrated a high proportion of referred patients with *possible* significant fibrotic NASH (now termed MASH).

Following diagnosis and in line with new guideline recommendations, fibrotic MASH patients in the present study were advised immediate lifestyle interventions, pharmacotherapy for metabolic and cardiovascular risk factors, and an appropriate surveillance program at our expert center. The patients were guided by a dietician for an optimal diet composition. Emphasis was placed on an individualized approach for each patient according to degree of fibrosis, capability, and preferences. Non-fibrotic MASH patients were advised general lifestyle interventions, optimized treatment of metabolic syndrome, and surveillance with non-invasive liver and fibrosis blood markers at primary non-expert centers [3].

The present study offers a unique evaluation of liver biopsy in all patients. Liver biopsy/histology is still the gold standard of MASLD and MASH. Non-invasive tests are expected to replace liver biopsy. However, the correlation between FIB-4 and degree of fibrosis is far from perfect, as shown in our study with considerable overlap between fibrosis stages. Complications to liver biopsy are rare and usually not serious, as also demonstrated in the present study. We believe that liver biopsies will still be relevant and used in an individualized medicine and case-by-case manner.

Prediction of advanced fibrosis is far from easy. Our study demonstrated that older age, the presence of diabetes type 2, and higher BMI are apparent risk factors for advanced fibrosis. In our study, hypertension, hypercholesterolemia, and gender per se were not associated with advanced fibrosis. For comparison, Rashu et al. [11] found type 2 diabetes to be a risk factor for significant fibrosis. The European NAFLD registry [12] aims to prospectively gather real-life data in a large cohort in order to increase knowledge about MASLD and better identify risk factors for advanced fibrosis. For now, the present study supports focus on a cardiovascular risk profile with emphasis on diabetes and elderly and obese patients.

Limitations of the study include the inherent selection bias for study population, and therefore, outcomes cannot be generalized as such. Also, the admission criteria may have favored the selection of more fibrotic patients. This was, however, a well-chosen selection in order not to include too many patients with simple MASLD. Further, not all patients had a FIB-4 score available. This became readily available in 2020, well into the study period.

## 5. Conclusions

We conclude that it is possible to identify a large proportion of patients with clinically significant fibrosis. The applied individualized medicine approach, including cardiometabolic–liver risk profile per recommendations in the new guidelines, together with our admission criteria, is useful for selecting criteria for identifying individual patients at high risk of fibrotic MASH and for referring patients to management at referral and expert care centers in a real-world setting at our clinic in Denmark and potentially more broadly. Our findings support the updated recommendations to healthcare providers for applying individualized medicine (case-finding) strategies for diagnosis and management of MASLD and especially fibrotic MASH patients [3,4].

## Figures and Tables

**Table 1:** Multivariate logistic regression for the prediction of significant fibrosis (F2–F4). Odds ratio > 1 indicates increased risk. Older age, (higher BMI), and presence of type 2 diabetes apparently increased the risk.

Variable	Odds Ratio	95% CI	*p*-Value
Hypertension	0.690	0.244–1.950	0.483
Age	1.049	1.008–1.092	0.018
Male gender	1.490	0.564–3.936	0.421
BMI	1.086	0.996–1.185	0.061
Hyperlipidemia	0.858	0.308–2.394	0.770
Type 2 diabetes	2.953	1.156–7.545	0.024

CI: confidence interval; BMI: body mass index.

## Data Availability

The original contributions presented in this study are included in the article. Further inquiries can be directed to the corresponding author.
